# Estrogen Receptors: A New Frontier in Alzheimer’s Disease Therapy

**DOI:** 10.3390/ijms25169077

**Published:** 2024-08-21

**Authors:** Giovanni Luca Cipriano, Emanuela Mazzon, Ivan Anchesi

**Affiliations:** IRCCS Centro Neurolesi “Bonino-Pulejo”, Via Provinciale Palermo, Contrada Casazza, 98124 Messina, Italy; giovanniluca.cipriano@irccsme.it (G.L.C.); ivan.anchesi@irccsme.it (I.A.)

**Keywords:** Alzheimer’s disease, estrogen receptors, estrogen signaling, neurodegenerative diseases

## Abstract

Alzheimer’s disease (AD) is a long-term neurodegenerative condition that leads to the deterioration of neurons and synapses in the cerebral cortex, resulting in severe dementia. AD is significantly more prevalent in postmenopausal women, suggesting a neuroprotective role for estrogen. Estrogen is now known to regulate a wide array of physiological functions in the body by interacting with three known estrogen receptors (ERs) and with the β-amyloid precursor protein, a key factor in AD pathogenesis. Recent experimental evidence indicates that new selective ER modulators and phytoestrogens may be promising treatments for AD for their neuroprotective and anti-apoptotic properties. These alternatives may offer fewer side effects compared to traditional hormone therapies, which are associated with risks such as cardiovascular diseases, cancer, and metabolic dysfunctions. This review sheds light on estrogen-based treatments that may help to partially prevent or control the neurodegenerative processes characteristic of AD, paving the way for further investigation in the development of estrogen-based treatments.

## 1. Introduction

At the cellular level, AD is marked by a progressive reduction of neurons and synapses in the cerebral cortex. This decline manifests as dementia, initially impairing memory before progressively affecting all cognitive functions [1]. Post-mortem neuropathological assessments reveal the spectrum and severity of the disease, showing characteristic cellular changes such as extracellular amyloid plaques, intracellular neurofibrillary tangles, and neuritic plaques [2,3,4]. These lesions progressively affect both cortical and subcortical areas, exacerbating clinical symptoms as they progress [5,6]. The higher prevalence of AD in postmenary and older women relative to men has been linked to some potent neuroprotective effects of estrogen treatments mediated through estrogen receptor (ER)-activated transcriptional machineries and the signal transduction by membrane ERs (mERs) [7]. Despite its close association with aging, AD is not an inevitable consequence of growing older. Surprisingly, it can also manifest in people in their 40s and 50s. The course of the disease can span from 2 to 20 years, often concluding with vulnerability to infections and other physical conditions that can accelerate and decline towards death [8].

The gradual onset of AD, often mistaken as part of natural aging, leads to misunderstandings about the disease. Symptoms like memory loss and confusion are often wrongly attributed to aging, overshadowing the distinct pathological progression of AD. This misconception persists partly because, unlike some illnesses, AD presently lacks preventive measures [9]. The risk factors and prevalence of AD are critical due to the absence of a cure. Various studies point to risk factors such as midlife obesity [10], diabetes [10], hypertension [11], smoking [12], depression [13], and low education levels as increasing the likelihood of developing AD [11]. Additionally, physical inactivity during midlife is as significant a risk factor as genetic predispositions like the APOE ε4 allele [14]. Notably, there is evidence that addressing these modifiable risk factors, including those affecting vascular health, may reduce AD risk [15,16]. Yet, other factors like insulin pathway dysregulation also play a significant role in increasing AD risk, highlighting the complexity of the disease’s etiology [17,18].

Estrogens have a larger scope of influence not only in the reproductive system but also on the physiological processes taking place in the brain, related to learning, memory, and neuroprotection against AD [19].

Many findings highlight the action of estrogens through their receptors, demonstrating spatiotemporal ERα and ERβ expression signaling decrement due to the aging of the brain [20,21,22]. This may lead not only to a loss of potential neuroprotection factors but also to the whole resilience of the organ, with marked sex differences involving female individuals of AD animal models [23,24]. The critical interplay between estrogen signaling and AD strongly matches with emerging data on estrogen receptor genes, cognitive decline, and AD dementia in postmenopausal women, giving a compelling narrative for the potential of estrogen as a neuroprotective agent [25]. These observations are also in line with the following observations: the use of hormone therapy is positively associated with regional brain volumes among women in general, regardless of cognitive status, and that hormone therapy indicates a protective role of estrogen against dementia, which might be modifiable by several factors such as lifestyle, timing of the hormone therapy, and genetic risk [26].

ERα is mostly expressed in regions of the brain such as the amygdala and hypothalamus based on the expression pattern. Its distribution is linked to the role of ERα in modulating autonomic and neuroendocrine responses, as well as emotional processing. The presence of ERα in these areas suggests that it plays a significant role in the estrogen-mediated regulation of stress responses, sexual behavior, and metabolic homeostasis. The importance of the involvement of ERα in these functions is further reached by its association with the serotonin and norepinephrine systems, both of which are implied in mood regulation and have also been associated with the pathophysiology of depression and other affective disorders [27].

On the other hand, ERβ shows a distinct distribution pattern, with high levels of expression in the hippocampus, entorhinal cortex, and cerebral cortex [20]. These regions are associated with higher cognitive functions, including learning, memory, and spatial navigation. The prevalence of ERβ in these areas suggests its involvement in the modulation of synaptic plasticity, neurogenesis, and neuronal survival, mechanisms that are fundamental to cognitive health and resistance to neurodegenerative diseases. The expression of ERβ in these brain areas shows its potential protective role against neurodegenerative conditions such as AD and highlights the therapeutic possibilities of targeting ERβ in cognitive disorders and brain injuries [28].

The differential expression and regulation of ERα and ERβ are not static throughout life. For example, epigenetic mechanisms, such as DNA methylation, play such an important role in dynamically regulating the expression of ERα during brain development and in response to environmental changes [29,30]. This regulation is crucial for the well-timed modulation of estrogen responsiveness, ensuring appropriate neuronal development and function. The dynamic regulation of ERα expression through epigenetic mechanisms highlights the complexity of estrogen signaling in the brain and its significance across different stages of life [31].

## 2. The Role of Estrogen Receptors in Alzheimer’s Disease: Molecular Mechanisms, Therapeutic Potential, and Biomarkers

An important class of ligand-activated transcription factors playing their critical roles in mediating the actions of estrogens within a cellular environment would be the estrogen receptors. These belong to the superfamily of nuclear hormone receptors and exist in two isoforms: ERα and ERβ, which are products of separate genes [32,33]. They mediate gene expression by binding to the estrogen response element on the DNA in the presence of estrogens, particularly estradiol [34]. This discovery greatly broadened the conceptual framework of estrogen action and set the stage for distinct ERα and ERβ actions in reproductive organs, bones, and brain, thereby reshaping drug discovery. There is also evidence showing that not only the reproductive tissues but also cardiovascular, immune, and central nervous systems are reliant on ERβ [35,36]. The widely divergent roles and complex interactions of such receptors are a testimony to their status as master regulators of physiological processes and critical therapeutic targets in a plurality of diseases, among them hormone-related cancers and cardiovascular diseases [37,38].

The significance of estrogen signaling pathways in the pathogenesis of dementia has also been studied through data-driven approaches. The estrogen signaling pathway is one of the seven key hormonal pathways converging at the core of the hormone interaction network associated with dementia and AD, along with a significant role of the insulin pathway in learning and memory, which are compromised in the progression of the disease. Translational validation of this model through off-target pharmacological effects and knockout mouse models has suggested substantial contributions of estrogen signaling pathways to dementia pathogenesis. Four key proteins (MAPK3, NOS1, CREB1, and JUN) representing different hormonal signaling pathways have been identified as potential biomarkers with measurable activities under AD conditions, indicating the mechanistic involvement of these pathways in AD dementia pathology [39].

Xing et al. studied the roles of nuclear receptors, including ERα, ERβ, and the membrane receptor mER (also known as GPR30), in mediating the effects of estrogen on hippocampal synaptic plasticity through the mammalian target of the rapamycin complex 2 (mTORC2) pathway. This pathway is involved in actin cytoskeleton polymerization and long-term memory. Treating animals with nuclear receptor antagonists (MPP/PHTPP) or the mER antagonist (G15), alone or in combination with A-443654, an activator of mTORC2, researchers examined changes in hippocampal SRC-1 expression, mTORC2 signaling, actin polymerization, synaptic protein expression, CA1 spine density, and synapse density. Antagonism of nuclear receptors or mER significantly reduced most parameters, except for synaptophysin expression. Activation of mTORC2 significantly reversed these reductions, except for changes in SRC-1, rictor, and synaptophysin expression. These findings suggest that both nuclear receptors and mER similarly contribute to alterations in structures and proteins associated with synaptic plasticity. mTORC2 thus emerges as a potential novel target for hippocampal-dependent dementia and AD treatment [40].

Xiong et al. studied how estrogen receptors ERα and ERβ differently influence tau phosphorylation and their effects on AD through the miR-218/PTPA pathway. They employed specific plasmids designed to overexpress ERα and ERβ, which were then transfected into HEK293/tau cells. Additionally, RNA interference (RNAi) approaches were used for the knockdown of ERα, ERβ, and PTPa to investigate their roles and also to show the enhancement of ERα and the reduction in tau phosphorylation. This was further supported by experiments using a non-specific ER antagonist, ICI 182780, and specific shRNA plasmids targeting mouse ERα or ERβ. Examination of tau aggregation in HEK293/tau cells upon ERα or ERβ overexpression showed that ERα increased tau hyperphosphorylation by elevating miR-218 levels, which decreased PTPa levels. Conversely, ERβ overexpression resulted in decreased tau phosphorylation. These results indicated that ERα caused an abnormal tyrosine hyperphosphorylation of glycogen synthase kinase-3β and protein phosphatase 2A, which are the major tau kinase and phosphatase, respectively. In contrast, ERβ inhibited tau phosphorylation by limiting miR-218 levels and restoring normal phosphorylation balance. This study concluded that ERα and ERβ have opposite effects on tau phosphorylation in AD, suggesting they could be targets for AD therapy via the miR-218/PTPA pathway [41].

ERα is found to co-localize with neurofibrillary tangles (NFTs) in the AD brain, indicating a potential role in AD pathology. There is no significant difference in ERα expression between AD and control brains, suggesting that the presence of ERα in NFTs is a specific pathological feature rather than a result of overall altered ERα expression. ERα interacts with tau protein in vivo, and this interaction is increased in AD, possibly contributing to the sequestration of ERα in NFTs. Overexpression of tau protein inhibits the transcriptional activity of ERα, potentially affecting estrogen signaling and its neuroprotective roles in AD. These findings suggest a mechanism where the sequestration of ERα by tau pathology might contribute to the loss of estrogen-mediated neuroprotection in AD, offering insights into gender differences in AD prevalence and progression [42].

Mesa-Herrera et al. aimed to evaluate estrogen receptor signalosome proteins as early biomarkers in prodromal AD, independent of amyloid-β production and tau phosphorylation. They classified fifty-three participants into cognitively normal healthy controls (HC), mild cognitive impairment (MCI), and subjective memory complaints (SMC) groups. Levels of estrogen receptor signalosome proteins, amyloid-β (Aβ), total tau (t-tau), and phosphorylated tau (p-tau) in cerebrospinal fluid (CSF) were analyzed using univariate, bivariate, and multivariate statistical analyses. Their results indicated that components of the estrogen receptor signalosome, such as caveolin-1, flotillin-1, estrogen receptor alpha (ERα), insulin growth factor-1 receptor beta (IGF1Rβ), prion protein (PrP), and plasmalemmal voltage-dependent anion channel 1 (VDAC), were detectable in the CSF of all subjects across the HC, MCI, and SMC groups. These components were elevated in MCI subjects and slightly increased in SMC subjects compared to HC subjects, suggesting early modifications in nerve cells. Using a multivariate approach, it was found that the combination of ERα, IGF-1Rβ, and VDAC are the main determinants of group segregation with sufficient resolution to predict the MCI stage. Most statistical analyses revealed no significant relationships or interactions with classical AD biomarkers at either disease stage. The conclusion was that alterations in the estrogen receptor signalosome might provide useful diagnostic information on preclinical stages of AD independently from classical biomarkers. The dynamic changes of these potential biomarkers in the CSF could help understand the biochemical processes underlying early pathology associated with AD [43]. Other authors have similarly found that CSF levels of Aβ1–42 are significantly reduced in patients who convert to AD within 5–10 years, making it a reliable early biomarker. Total tau (T-tau) and phosphorylated tau (P-tau) levels are elevated closer to the onset of AD, indicating their roles as later markers [44]. Furthermore, for better diagnosis and prognosis, blood biomarkers, including plasma Aβ1–42 and the Aβ42/40 ratio, have shown promising features in differentiating prodromal and clinical AD, offering a less invasive and more cost-effective alternative to CSF biomarkers or PET imaging [45].

To investigate cell type-specific transcriptional changes and their associated pathways in AD, Wang et al. performed an integrated analysis using 4441 differentially expressed genes (DEGs) derived from cortical samples of single cells via single-nucleus RNA sequencing (snRNA-seq) of subjects with AD pathology and healthy controls from three studies. DEGs in microglia, astrocytes, oligodendrocytes, excitatory and inhibitory neurons, and endothelial cells were analyzed. Common DEGs across three studies were identified, which also overlapped in at least two studies. Their analysis showed that cell type-specific alterations in dysfunctional pathways involved in AD pathogenesis, including the estrogen signaling pathway, result as the most frequently dysregulated across cell types in AD. A further AD-related loss of estrogen receptors in cells impacts neuronal function and brain stability, suggesting how restoring estrogen signaling or improving it could be a promising treatment for AD [46].

Ishunina et al. investigated the impact of the ERα splice variant TADDI on neuronal size and its association with AD and pneumonia in the human supraoptic nucleus (SON). Using post-mortem brain tissue from 58 control patients aged 20 to 94 years and 26 AD patients aged 54 to 94 years, they analyzed neuronal morphometric parameters in relation to TADDI expression. Regardless of gender, age, or AD status, SON neurons with moderate to strong TADDI staining exhibited significantly smaller nuclear and perikaryal sizes compared to neurons lacking this ERα splice variant expression. Conversely, neuronal nuclei and perikarya were larger in SON neurons with moderate and strong nuclear staining for wild-type ERα compared to neurons without this receptor, indicating a potential stimulatory effect of classical ERα on neuronal metabolic activity. Key differences between AD patients, particularly female patients, and healthy controls include generally larger neuronal sizes in AD patients despite the presence of TADDI. This suggests a potential compensatory metabolic activation in response to neurodegenerative damage, further substantiated by increased TADDI immunoreactivity in control patients with pneumonia or respiratory insufficiency, suggesting that TADDI expression might be related to hypoxic conditions. The reduction of morphometric parameters measured in SON neurons related to the presence of the TADDI splice variant implies that this ERα variant exerts an inhibitory role on neuronal metabolic activity, contrary to the stimulating effects mediated by canonical ERα. TADDI expression has been linked with pneumonia or respiratory insufficiency in the control group, suggesting the possibility that TADDI might be involved in the body’s response to hypoxic conditions and is also related to neuronal shrinkage in AD degenerative processes [47].

Such studies pointed out the critical effect of estrogen receptors on AD pathology and their therapeutic potential. The influence of nuclear and membrane estrogen receptors on hippocampal synaptic plasticity during mTORC2 signaling is shown. It can be considered an essential pathway for memory and cognition, which may lead to AD therapy. The delineated effects of modulation of this pathway in relation to estrogen receptor-mediated AD treatment would be outlined. In addition, it has been shown that ERα and ERβ differentially modulate tau phosphorylation through the miR-218/PTPA pathway, indicating some specific targets for therapy. A significant interest given early diagnosis will also be the detection of estrogen receptor signalosome proteins as early biomarkers in the prodromal AD state, independent of both amyloid-β and tau. Taken together, these data argue for an essential role of estrogen pathways in AD and increased focus on estrogen signaling for further therapeutic targeting (Table 1).

## 3. Therapeutic Compounds Targeting Estrogen Signaling in Alzheimer’s Disease

### 3.1. Hormonal and Hormonal Replacement Treatments

The modulation of estrogen signaling using different pharmacological and natural compounds is crucial to not only better understand the interactions by themselves but also to comprehend the signaling properties of the receptors.

Interestingly, estrogen replacement in ovariectomized (OVX) female rats shows protective effects against status epilepticus (SE)-induced alterations in the hippocampal dentate gyrus transcriptome. It significantly reduces gene expression levels across major functional pathways in the SE-induced model, AD one included, implying a potential role for estrogen signaling in the mitigation of the AD-associated neurodegenerative processes. To better understand the molecular landscape of their model, Iacobas et al. performed RNA sequencing (RNA-seq) to analyze changes in the hippocampal transcriptome following SE in OVX rats with and without estrogen replacement. DEG and pathway analysis revealed significant alterations in genes and pathways related to AD, indicating estrogen’s protective role against these changes. KEGG was then used to identify commonly shared genes between estrogen signaling (ESG) and the neurotransmission pathways. These genes serve as the primary mediators through which ESG influences synaptic connections and brain circuitry. The common genes identified include adenylate cyclase (Adcy2, Adcy3, Adcy5, Adcy6, Adcy7, Adcy8, and Adcy9); oncogenes (Akt1, Ak3, Fos, Hras, Kras, raf1, and Src); transcription factors (Atf4 and Atf6b); calmodulins (Calm1, Calm2, and Calm3); membrane receptors (Gabbr1, Gabbr2, Grm1, Itpr1, and Itpr2); binding proteins (Creb1, Creb3l1, Gnai1, Gnai2, Gnai3, Gnao1, and Gnaq); ion channels (Kcnj3, Kcnj5, and Kcnj9), and kinases (Map2k1, Mapk1, Mapk3, Pik3ca, and Pik3cb). These genes were part of the complex transcriptomic regulation of hippocampal dentate gyrus neurotransmission by estradiol (E2) replacement, which is functionally important for preserving neuronal plasticity following SE. These findings imply that estrogen signaling may act as a shield against SE-induced alterations in neurotransmission, potentially contributing to the maintenance of brain function in postmenopausal women, highlighting estrogen’s neuroprotective roles beyond its traditional reproductive functions, with implications for AD [48].

Further supporting the neuroprotective role of estrogens, Morelli et al. used murine models to demonstrate the involvement of estrogens in neuroprotection. They examined young human brain cells from the nucleus basalis of Meynert (hfNBM) and their role in memory improvement in AD animal models. Their study involved examining the appearance, electrical behavior, and response to substances like NGF and estrogen of these cells. When these hfNBM cells were introduced to rats with brain damage, the rats showed improved memory skills, suggesting the potential of estrogen and NGF in enhancing memory and cognitive function in AD [49].

The landscape of Alzheimer’s disease (AD) symptoms is intricately linked to olfactory neural loss due to neurodegenerative mechanisms. A study by Pooley et al. explored the influence of 17-β estradiol on neurite outgrowth in mouse olfactory epithelium (OE) cultures, focusing on the role of estrogen receptor alpha (ERα). Treatment with estradiol significantly enhanced neurite outgrowth compared to the vehicle. Both ERα and ERβ were expressed in OE cultures, and their expression levels increased following estrogen treatment. Notably, the neurite outgrowth effect was replicated by an ERα-specific agonist but not by an ERβ agonist, indicating that estradiol enhances neurite outgrowth primarily through ERα. These findings suggest that estradiol may help improve olfactory deficits in neurological disorders related to estrogen deficiency by promoting olfactory neuron regeneration through ERα, emphasizing the roles of estradiol and ERα in neural repair, and suggesting potential treatments for olfactory dysfunction and related conditions [50].

Interestingly, androgens and estrogens interact in different ways in the molecular context of cell viability, showing both neuroprotective and neurotoxic effects. Using the N27, PC12 neuronal, and C6 glial cell lines derived from rats, the research aimed to elucidate the mechanisms underlying the effects of these hormones on cells exposed to oxidative stress. Neither androgens nor estrogens offered protection against oxidative stress in glial cells. However, in neuronal N27 and PC12 cells, these hormones exerted neuroprotective properties through the activation of estrogen receptors. Interestingly, temporary hormone deprivation negated the protective effects of sex hormones against oxidative stress. Conversely, when hormones were introduced after oxidative stress exposure, they exacerbated the damage in both neuronal and glial cells. Sex hormones could either protect against or exacerbate oxidative stress-induced cell damage depending on the cell type, cellular environment, and the hormone exposure timing related to the oxidative stress event. These findings suggest that the protective effects of sex hormones are mediated through estrogen receptors in a healthy neuronal environment, but in a high oxidative stress condition, they can worsen cell loss, which is a fundamental characteristic to monitor in AD [51].

Focusing on the molecular and signaling landscape related to estrogen receptors, a study by Amaro et al. investigated the role of lipid rafts and their signalosome in neurons. Their research explored how menopause and subsequent estrogen deficiency affect AD onset and progression and whether it is possible to act to prevent it with substitutive therapies. Using immunoblotting, immunoprecipitation assays, and lipid analyses on frontal cortex samples from pre-menopausal, post-menopausal, and AD-affected subjects, their results demonstrated distinct patterns of membrane estrogen receptor alpha (mERα)-related signalosomes during menopausal stages and in AD. It was found that mERα dissociates from the voltage-dependent anion channel (VDAC) and caveolin-1, leading to progressive VDAC dephosphorylation, which enhances neurotoxicity. Alterations in lipid profiles and percentages in lipid rafts suggest increased liquid-ordered phases in AD compared to controls. These findings indicate that menopause, along with disturbances in lipid raft structure and function, contribute to AD onset and progression, and the correct and physiological activation induced by 17β-estradiol used as replacement therapy could improve and prevent these particular damaging events [52].

In the context of hormone therapy, Boyle et al. investigated how self-reported hormone therapy (HT) use affects brain volume in elderly women aged 71–94 years with mixed cognitive status using various factors, including lifestyle, timing of HT use, and genetic risk. Using MRI data, demographic information, cardiovascular disease (CVD) risk factors, and APOE4 status, their study demonstrated, regardless of cognitive status, that HT use was positively associated with regional brain volumes changes, where certain brain regions showed larger volumes. Estrogen therapy in postmenopausal women is correlated with brain volumes, which are essential for cognitive function, thus emerging as a potential therapeutic strategy for elderly individuals [53].

Another complex issue regarding the role of estrogens in women with a more consistent role in a postmenopausal scenario is the role of lipid dysmetabolism and its consequences. Investigating the effects of warning levels of estrogen and its receptors on cognitive impairment and dyslipidemia in postmenopausal women is crucial to establishing how the use of hormone supplementation or lipid-lowering compounds could counteract these effects. Using both in vivo (LDLR^−/−^ mice with ovariectomy) and in vitro experimental setups to demonstrate that the decrease in estrogen and estrogen receptors was able to accelerate dyslipidemia and cognitive deterioration. Treatment with either β-estradiol or simvastatin improved lipid profiles and cognitive functions. Hence, therapeutic interventions involving enhanced levels of estrogen or altered lipid metabolism may reverse these effects. This study indicated that a strong relationship between estrogen receptors and AD gives postmenopausal women a possible way to slow disease progression [54].

### 3.2. Phytocompounds

Phytocompounds, the bioactive compounds found in plants, play a crucial role in modern medicine. These natural substances, containing a plethora of intriguing bioactive compounds, have been utilized for centuries in traditional medicine systems. In recent years, the scientific community has increasingly recognized the potential of phytocompounds to contribute to contemporary healthcare. Their diverse therapeutic properties make them valuable candidates for developing new drugs and treatments, which could be implemented in the future for AD and dementia treatment. Here, we describe how their administration and activity affect estrogen signaling and their peculiar properties.

For instance, the curative effect and possible mechanism of Acori graminei rhizoma on AD have been studied to understand how its active biomolecules interact in the context of AD. The analysis showed the involvement of several genes in AD and their links to estrogen signaling. The key genes identified included APP, CASP3, MAPK1, MAPT, VEGFA, ACHE, GSK3B, ESR1, and DRD2, among others. These genes are implicated in various AD-related pathways, such as the AD pathway, serotonin synapse, and estrogen signaling pathway. The mutual link between the AD estrogen signaling pathways involves core targets like ESR1 (Estrogen Receptor 1) and GSK3B, indicating that estrogen signaling may influence the development or progression of AD. As stated previously, the involvement of Chinese medicine plants used in common practice seems to converge toward the interaction, among others, of estrogen signaling [55].

Wang et al. identified 1424 differentially expressed genes (DEGs) between AD and normal control tissues, with pathways such as neuroactive ligand–receptor interaction, estrogen signaling, and Notch signaling pathway significantly enriched. Key findings included the identification of common target genes across active ingredients in traditional Chinese medicine that might offer new insights into AD treatment. Specifically, HTR2A and ADRA2A were highlighted among the shared genes and were also enriched in the pathway of neuroactive ligand–receptor interactions, suggesting their potential involvement in AD’s molecular mechanisms and the therapeutic effects of estrogen signaling [56]. Exploring further in traditional Chinese medicine, the herbal formula Sagacious Confucius’ Pillow Elixir (SCPE) was investigated in an AD mouse model induced by D-galactose. SCPE was found to exert estrogenic effects in the brain by increasing estrogen levels and the expression of ERα receptors. This suggests that SCPE may ameliorate the cognitive decline associated with AD through pathways influenced by estrogen signaling. SCPE enhances synaptic structure plasticity, promoting BDNF release, actin polymerization, and coordinating cofilin activity. Additionally, SCPE enhances synaptic functional plasticity by increasing the density of postsynaptic density 95 (PSD95) proteins and the expression of the functional receptor AMPA. These findings indicate SCPE beneficial effects on cognitive function may rely on its influence on synaptic plasticity and architecture, linking estrogen signaling to the mitigation of AD symptoms [57].

Milk thistle flavonoids, such as Silibinin, could be implemented in the treatment of AD patients and are also relevant for the biology of estrogen receptors. Rats injected with Aβ1–42- and Aβ1–42-injected groups treated with various doses of Silibinin or donepezil, a standard AD medication, were studied to understand the neuroprotective potential and properties of this specific flavonoid, using high-performance liquid chromatography (HPLC) to confirm Silibinin brain availability, Western blotting for protein expression analysis, and immunohistochemical staining for caspase 3 to evaluate neuronal damage. The results showed that Silibinin significantly counteracted Aβ1–42-induced memory impairment and neuronal damage in the hippocampus. Specifically, Silibinin treatments improved performance, indicating a recovery of spatial and recognition memory. Furthermore, the treatment reduced caspase-3 expression levels, exhibiting decreased neuronal apoptosis. From a molecular approach, Silibinin modulated the ER pathway, down-regulating ERα and ERβ expressions previously altered by Aβ1–42 treatments and also acting on mitogen-activated protein kinases (MAPKs) and the PI3K-Akt pathway, suggesting a complex interplay of neuroprotective mechanisms. Thus, Silibinin offers a potential therapeutic effect against AD by mitigating Aβ1–42-induced cognitive deficits and neuronal damage, partly through the modulation of estrogen receptors and related signaling pathways [58]. Another phyto-related molecule, Puerarin, isolated from Pueraria lobata, has been investigated for its neuroprotective properties against Aβ1–42-induced toxicity in primary cortical neurons. Focusing on the role of ER activation, particularly ERβ, the treatment of primary cortical neurons with Puerarin prior to Aβ1–42 exposure assessed cell viability, neurite outgrowth, and ER expression levels. Puerarin markedly suppressed Aβ1–42-induced neuronal death and promoted neurite growth in a dose-dependent manner. The neuroprotective effects of Puerarin were reversed by ER blockade, more so with ERβ than ERα. Further investigations confirmed the dependence of Puerarin’s protective effects on ERβ activity. Puerarin counteracted Aβ1–42-induced reduction in ERβ levels and caspase cleavage via ERβ, emphasizing Erβ’s central role in mediating Puerarin’s neuroprotective actions. This finding not only emphasizes the importance of ERβ in neuronal protection but also suggests Puerarin as a promising phyto-estrogenic compound for therapeutic applications in neurodegenerative diseases, providing a viable alternative to traditional estrogen replacement therapy with fewer side effects [59].

Calycosin, a phytoestrogen derived from Radix Astragali, was investigated for its cognitive benefits in a transgenic mouse model of AD. Intraperitoneal injections of various doses of Calycosin in these mice reduced hippocampal beta-amyloid, Tau protein, interleukin-1beta, tumor necrosis factor-alpha, acetylcholinesterase, and malondialdehyde levels while also increasing acetylcholine and glutathione activities. Calycosin’s protective effects, including improved cognitive ability and anti-oxidative and anti-inflammatory effects, were mediated through the activation of the protein kinase C (PKC) pathway, disrupted by a PKC inhibitor, calphostin C. This suggests that Calycosin mitigates oxidative stress and inflammatory responses in the hippocampus of AD model mice, leading to improved cognitive function [60].

Geniposide, a component of Tongluojiunao (TLJN), a Chinese herbal medicine, activates the non-classical estrogen signaling pathway. Estrogen’s importance in maintaining neuronal function and its protective effects against neurodegeneration are well-documented, but the adverse side effects of estrogen therapy have led to interest in phytoestrogens like Geniposide. Geniposide’s protective effect against Aβ1–42-mediated neuronal death in cultured hippocampal neurons involves the PI3K and MAPK pathways, suggesting that it functions as a phytoestrogen. This provides a potentially safer alternative for neuroprotection in AD without the risks associated with direct estrogen therapy [61].

Young coconut juice (YCJ) also seems to exert an estrogen receptor modulation effect against AD pathology in orchidectomized (orx) rats. Tissues from these rats have been analyzed for histopathological changes associated with AD, specifically examining neurofilament 200 (NF200), parvalbumin (PV), beta-amyloid (Aβ), and estrogen receptors (ERα and ERβ) through immunohistochemical staining. The results showed that orx rats exhibited a significant reduction in NF200- and PV-reactive neurons in the hippocampus and cerebral cortex, in parallel with an increment in Aβ-containing neurons. Treatment with Estradiol Benzoate (EB) or YCJ neutralized these effects in a dose-dependent manner, suggesting that the active components of YCJ are able to interact with estrogen receptors in the brain. Notably, a significant correlation was observed between NF200-/PV-reactive neurons and ERα-/ERβ-reactive neurons, suggesting the mechanism through which YCJ may exert its protective effects against AD pathology [62].

The studies reported here highlight the potential of phytocompounds to interact with estrogen receptors and their signaling pathways, providing neuroprotective effects and offering promising future therapeutic strategies to implement for AD treatment.

### 3.3. Synthetic and Non-Hormonal Estrogenic Modulators in Alzheimer’s Disease Therapy

Based on the other observations reported in this review, the route of synthetic compounds and non-hormonal molecules needs to discussed to better comprehend the plethora of interesting effects that can be archived through the possible future usage of these molecules.

Park et al. developed and evaluated boronate ester-based pro-estrogens for selective ERβ modulation, leveraging hydrogen peroxide (H_2_O_2_) activation in contexts of neurodegenerative and inflammatory diseases characterized by elevated reactive oxygen species (ROS) levels. Their methodology involved synthesizing boronate ester derivatives of the potent ERβ agonist diarylpropionitrile (DPN) and endogenous estrogens, assessing their estrogen receptor binding affinities, H_2_O_2_-mediated conversion kinetics to phenols, ROS selectivity, and ERβ transcriptional activation in cells. Their results demonstrated that boronate esters exhibited lower ERα and ERβ affinities and were rapidly converted into active phenols via H_2_O_2_, showing preference for H_2_O_2_ over other ROS. The most promising compound, a boronate ester-masked DPN derivative, had reduced binding affinity but effectively converted to DPN in the presence of pathological H_2_O_2_ levels, restoring ERβ agonist activity in a cellular model. Thus, boronate ester pro-drugs could serve as promising molecular candidates for ER modulation in oxidative stress diseases by both neutralizing H_2_O_2_ and releasing ERβ-specific ligands, suggesting their potential for developing targeted estrogens or anti-estrogens [63].

Fekete et al. explored the consequences of chronic amyloid-β1–42 oligomer (AβO) administration on microglial responses and transcriptome-level inflammatory signatures in the rat hippocampus. Using a model of middle-aged rats, they administered intracerebroventricular infusions of AβO or a vehicle for four weeks, followed by treatment with artificial CSF or MCC950 (a NLRP3 inflammasome inhibitor) for an additional four weeks. AβO infusion induced NF-κB-triggered microglial activation and a sustained inflammatory response, characterized by elevated expression of pattern recognition and phagocytic receptors. Despite the absence of detectable Aβ1–42 plaques, likely due to microglial clearance of infused oligomers, this study observed an upregulation of neuronal inhibitory ligands and corresponding microglial receptors, alongside a downregulation of genes encoding estrogen receptor alpha (Esr1) and voltage-gated sodium-channel Na(v)1.1 (Scn1a). These molecular alterations were associated with impaired hippocampus-dependent spatial memory, linked to early clinical neurological changes observed in AD. The MCC950 application attenuated AβO-evoked microglial reactivity, restored the expression of neuronal inhibitory ligands, reversed the downregulation of ERα, and ameliorated memory impairments. These insights emphasize the chronic environmental risk posed by Aβ oligomers for neurodegenerative disorders like AD and the potential of targeting NLRP3 inflammasome pathways for therapeutic interventions, which appear to be linked to the estrogen landscape [64].

In their study, Imamura et al. focused on their investigation of the molecular pathways responsible for donepezil, an acetylcholinesterase inhibitor developed for the treatment of AD, induced differentiation of miPSC-NSCs into mature oligodendrocytes. To achieve this, the effect of donepezil was studied via the use of cell-based reporter gene arrays to determine the molecular pathway activated under differentiating conditions. Since the ability of donepezil to induce the activation and consequent transcriptional activity of ERE and the presence of ERα and Erβ in MBP-positive mature oligodendrocytes, the authors also used the estrogen receptor antagonist ICI 182780 to assert the relative contribution of both receptors in this molecular process. Using silencing systems based on siRNA decreased the number of MBP-positive oligodendrocytes that were upregulated by donepezil. This intriguing research shows how donepezil-induced oligodendrogenesis is fundamentally linked and carried on by the ERα and ERβ signaling pathways [65].

Other interesting findings about estrogen-regulating activities are related to the already aforementioned ICI 182780. Better known as Faslodex, its action has been studied against AD-associated neurodegenerative conditions. ICI 182780, in a concentration-dependent manner, was found to significantly promote neuron survival following the exposure to excitotoxic glutamate or β-amyloid, showing similar neuroprotective efficacy to the endogenous 17β-estradiol. ICI 182780 is able to directly induce a series of rapid intracellular Ca2+ variations comparable to 17β-estradiol, along with a sustained activation of extracellular signal-regulated kinase 1/2 (ERK1/2) and Akt (protein kinase B) and increased expression of spinophilin and Bcl-2, which indicate dendritic spine activity. These results support the potential of using neuroselective estrogen receptor modulators as safe alternatives to the traditional estrogen therapy toward post-menopausal cognitive decline and late-onset AD [66].

Kwakowsky et al. investigated the therapeutic potential of Estren, a non-classical estrogen signaling activator, in AD-associated cognitive decline and cholinergic damage. Estren mimics the rapid effects of estradiol on cell signaling pathways without engaging classical genomic pathways, avoiding estrogenic side effects. In mice, Estren restored cholinergic cortical projections and attenuated Aβ1–42-induced learning deficits, demonstrating its potential for treating cholinergic damage in AD without the adverse effects of traditional estrogen therapies. Estren’s neuroprotective effects are mediated through rapid activation of intracellular signaling pathways in BFC neurons, particularly the phosphorylation of CREB and ERK1/2 via ERα. This selective activation of non-classical estrogen signaling pathways offers a novel approach to AD treatment, targeting the cholinergic deficit preceding cognitive decline [67].

Another interesting molecular modulation of estrogen receptors, such as the nuclear ones, has been advanced by Wnuk et al. They aimed to explore the therapeutic potential of PaPE-1, a newly designed compound to selectively activate non-nuclear estrogen receptors, in the context of AD. In order to understand the potential of this compound, these researchers treated mouse neocortical neurons with Aβ-inducing apoptosis, loss of mitochondrial membrane potential, activation of caspase-3, induction of apoptosis-related genes and proteins, and increased levels of both reactive oxygen species (ROS) and lactate dehydrogenase (LDH). The results emerging from the treatment with PaPE-1 indicated that PaPE-1 is able to inhibit Aβ-induced neurotoxicity and apoptosis. PaPE-1 also reduced neurotoxicity, oxidative stress, and apoptosis by downregulating Aβ-induced FasL/FAS expression and upregulating Aβ-induced FasL. Interestingly, it was able to normalize Aβ-induced loss of mitochondrial membrane potential and restore the BAX/BCL2 ratio, indicating that PaPE-1 capacity significantly relies on the inhibition of the mitochondrial apoptotic pathway. These results suggest that PaPE-1 protects brain neurons by modulating the internal mitochondrial pathway against Aβ-induced toxicity and reducing oxidative stress and apoptosis [68].

Another study carried out by Gray et al. aimed to evaluate the neuroprotective properties of STX, a ligand acting as new selective estrogen receptor modulators (SERMs) for membrane estrogen receptors under Aβ exposure. Using the MC65 and SH-SY5Y neuroblastoma cell lines and primary hippocampal neurons from wild-type and Tg2576 mice, these researchers investigated STX properties, which resulted in a cell death reduction, mitochondrial dysfunction, dendritic simplification, and synaptic loss induced by Aβ. The authors also noted STX prevented Aβ-induced cell death in neuroblastoma cell lines, restoring the Aβ-triggered ATP level depletion and the mitochondrial gene expression. Notably, STX also increased ATP content and mitochondrial gene expression both in control neuroblastoma cells and primary neurons, regardless of Aβ presence. Furthermore, STX treatment enhanced dendritic spine arborization and densities in wild-type neurons and counteracted the reduced dendritic outgrowth caused by Aβ exposure in Tg2576 neurons. STX could act as a strong neuroprotective agent against Aβ toxicity, improving mitochondrial function while also promoting dendritic growth and synaptic differentiation [69].

Even molecules known for their effect on growing muscle and increasing muscle properties, such as the androgenic steroid 17β-trenbolone, have an effect on amyloid beta Aβ42 accumulation, apoptotic processes, and consequent neurodegeneration. In order to understand more about its role and pathophysiological effects, it was administered in adult and pregnant rats, as well as in primary hippocampal neurons. Its distribution and accumulation in the serum and mainly in the hippocampus were measured by a variety of methods, including liquid chromatography/mass spectrometry (LC-MS/MS), enzyme-linked immunosorbent assays (ELISAs), and Western blot analysis. 17β-trenbolone was shown to be mainly accumulated in the hippocampus of rat brains as well as the fetal brain, indicating its ability to cross both the blood–brain and placental barriers. It also altered serum hormone levels, induced apoptosis in primary hippocampal neurons, decreased presenilin-1 protein expression, increased caspase-3 activity, and promoted Aβ42 production. These outcomes suggest a potential contribution to neurodegenerative processes. Both the androgen and estrogen receptors resulted to have mediated the effects of 17β-trenbolone, pointing out the critical role of these in the reported neurotoxic effects and highlighting the importance of monitoring and avoiding the use of 17β-trenbolone for the possible development of neurodegenerative diseases [70].

In order to better understand how estrogen exerts its role in AD, the use of Aroclor1254, which belongs to the endocrine-disrupting chemicals (EDCs), helps find further mechanisms underlying AD and the role of estrogen signaling in offering neuroprotection against it. What was found is that estrogen, through the activation of estrogen receptors, particularly ERα, is able to mitigate the neurotoxicity induced by beta-amyloid and decrease the phosphorylation of tau protein in JNK signaling pathway-dependent mechanisms. Aroclor1254’s anti-estrogenic properties are able to increase beta-amyloid toxicity and tau phosphorylation by interfering with estrogen receptor signaling. This highlights the importance of estrogen signaling in defending against beta-amyloid-induced neuronal damage and suggests that the disruption of this signaling may contribute to the development and progression of AD [71].

**Figure 1:**
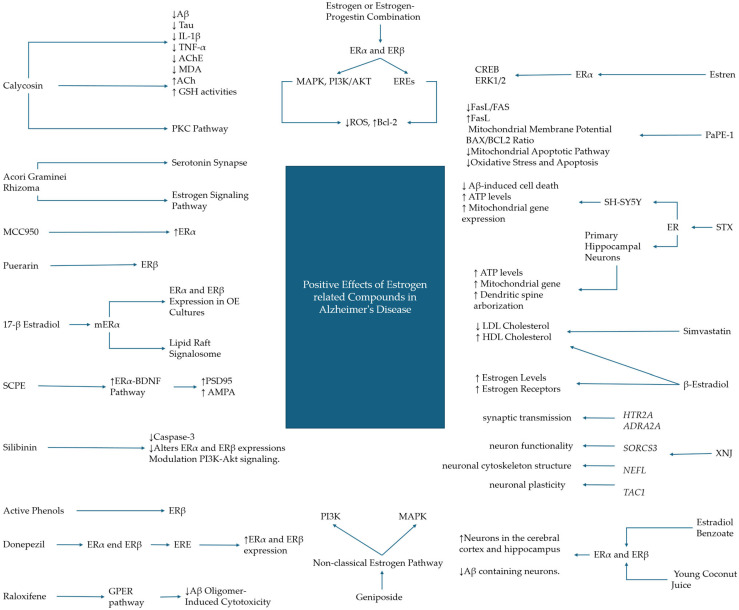
The neuroprotective effects and benefits exerted by molecules related to the activation of estrogen receptors and their signaling mechanisms in the context of Alzheimer’s disease.

The effects of estrogen receptors certainly need to be further investigated in women, in which the effects of the hormone and its related signaling are physiologically more relevant than in men. In this regard, the use of Raloxifene, a selective estrogen receptor modulator, which is known for its protective effects in liver and bone tissue without increasing estrogen-dependent tumor risks, was investigated in women since the higher prevalence of AD patients. Raloxifene mitigated Aβ oligomer-induced cytotoxicity effects in neuronal cells thanks to the mediation of the G protein-coupled estrogen receptor (GPER). The oligomers, associated with AD, induce neurotoxicity by damaging nerve membranes, and since women undergo post-menopausal declines in estrogen levels, a model involving SH-SY5Y neuroblastoma cells has shown the effects of Raloxifene and estradiol on Aβ oligomer-induced cell viability, oxidative stress, membrane lipid peroxidation, and intracellular calcium levels. The findings demonstrated that Raloxifene significantly improves cell viability, reduces oxidative stress markers, and prevents calcium influx induced by Aβ oligomers, comparably with estradiol but more related to Raloxifene. The protective outcomes offered by Raloxifene administration diminished upon pre-treatment with a GPER antagonist, suggesting the direct involvement of GPER in the mediation of its neuroprotective action [72] (Figure 1 and Table 2).

## 4. Conclusions

This review gathers solid information about the topic, and it is focused on emerging therapies and biomarkers, which are well aligned with the ongoing efforts of AD research. Supporting the involvement of estrogen and the activation of its receptors by novel pharmacological approaches is thus fundamental to understand the pathogenesis, progression, and also future possible treatment of the pathology. The localization of ERα and ERβ in different brain regions and their distinct roles in cognitive processes draw attention to targeted therapies toward specific estrogen receptor activation for symptom relief of AD. Estrogen replacement therapies, selective estrogen receptor modulators, and phytoestrogens appear as promising therapeutic options, with potential fewer obstacles in terms of neuroprotection, along with a better profile of adverse effects compared to traditional hormone therapies. Indeed, they should be further investigated to choose the best options to implement in the future of estrogen receptor activation molecules. This review highlights the existence of a plethora of compounds that are able to act in the estrogen molecular pathways. Further research in this peculiar field should be focused on the deep understanding of the molecular mechanisms of estrogen-mediated neuroprotection, and estrogen-based therapeutics should be designed focusing on the improvement of the cognitive condition in subjects at risk or already suffering from AD.

## Figures and Tables

**Table 1:** Studies that summarized the role of estrogen receptors in Alzheimer’s disease.

Experimental Model	Biological Specimen	Pathway	Compound/Therapy	Ref.
In silico	-	Estrogen signaling pathway and insulin signaling pathway	-	[39]
Adult female C57/BL6 mice (in vivo and in vitro)	hippocampus	ERs in hippocampal synaptic plasticity, particularly examining the mTORC2 pathway’s involvement in actin polymerization and synaptic protein expression	ER antagonists (MPP, PHTPP for nERs; G15 for mER) and the mTORC2 activator A-443654	[40]
18-month-old male Tg2576 mice (in vivo and in vitro)	HEK293/tau cells and mouse N2a cells	miR-218/PTPa pathway	ICI 182780	[41]
Human postmortem brain tissues and M17 human neuroblastoma cells (in vitro)	Hippocampal and cortical tissues	estrogen signaling pathway	-	[42]
Cohort of human subjects categorized into three groups: cognitively normal healthy controls (HC), mild cognitive impairment (MCI), and subjective memory complaints (SMC) (Clinical samples)	CSF samples collected from the participants	The pathways related to cellular signaling and neuronal survival, including interactions involving proteins like ERα, IGF-1Rβ, and VDAC	-	[43]
snRNA-seq (clinical samples (in vitro))	Cortex samples from 42 AD pathology subjects and 39 normal controls	Immune response and neuroinflammation Mitochondrial dysfunction across various cell types Estrogen signaling pathway disruption Oxidative stress response	-	[46]
58 control patients aged 20–94 years without neurological or psychiatric disorders, and 26 patients with AD aged 54–94 years. (clinical samples (in vitro))	Hypothalamic sections containing the SON were sourced from the Netherlands Brain Bank	Estrogen receptor signaling, particularly involving a splice variant of ERα, known as TADDI, and its effect on neuronal morphology and metabolic activity in the SON	-	[47]

ERs: estrogen receptors; mTORC2: mechanistic target of rapamycin complex 2; MPP: methyl-piperidino-pyrazole, an estrogen receptor antagonist; PHTPP: 4-phenyl-2,4,6-trimethylphenoxy-acetic acid, an estrogen receptor antagonist; nER: nuclear estrogen receptor; mER: membrane estrogen receptor; Tg2576: transgenic mouse model of Alzheimer’s disease; HEK293: human embryonic kidney 293 cells; N2a: mouse neuroblastoma 2a cells; PP2: specific inhibitor of Src kinase; ERα: estrogen receptor alpha; AD: Alzheimer’s disease; MCI: mild cognitive impairment; SMC: subjective memory complaints; CSF: cerebrospinal fluid; IGF-1Rβ: Insulin-like growth factor-1 receptor beta; VDAC: voltage-dependent anion channel; snRNA-seq: single-nucleus RNA sequencing; SON: supraoptic nucleus; TADDI: estrogen receptor alpha splice variant.

**Table 2:** Studies that summarized the involvement of compounds modulating estrogen signaling and their therapeutic implications in Alzheimer’s disease.

Experimental Model	Biological Specimen	Pathway	Compound/Therapy	Type of Compound	Ref.
Female Sprague Dawley rats, 8–9 weeks old, OVX (in vivo and in vitro)	Hippocampal DG tissue	Interaction and protection of synaptic transmission pathways by E2, including glutamatergic, GABAergic, dopaminergic, cholinergic, and serotonergic pathways. ESG significantly engaged.	17β-estradiol benzoate	Hormonal and Hormonal replacing treatments	[48]
in vivo and in vitro	Human cholinergic neurons isolated from the NBM of 12-week-old human fetuses. Male Wistar rats subjected to a NBM lesion induced	Nicotinic and muscarinic receptors; NGF/TrkA and estrogens	NGF and 17-β-estradiol	Hormonal and hormonal replacement treatments	[49]
Olfactory epithelium culture (in vivo and in vitro)	Rat olfactory epithelial cells	Estrogen receptor alpha pathway	17-β estradiol	Hormonal and Hormonal replacement treatments	[50]
N27 and PC12 neuronal cell lines C6 glial cell line (in vivo and in vitro)	Female rat-derived N27 cells Male rat-derived PC12 and C6 cells Human hippocampal tissue	Estrogen receptor pathway Membrane-associated AR45 pathway Oxidative stress pathway mediated by H_2_O_2_	Testosterone 17β-estradiol	Hormonal and hormonal replacement treatments	[51]
Human brain samples from three groups: premenopausal women (<50 years old), postmenopausal women (>65 years old), and AD patients (ADV/VI stages) (in vitro)	Human frontal cortex tissue.	ERα signalosome pathway, involving interactions with IGF-1Rb and VDAC1	17β-estradiol	Hormonal and hormonal replacement treatments	[52]
562 females: Patient with cognitive impairment (MCI and AD: 137 cognitively normal controls: 425 (clinical model)	Brain structure and volume, as assessed using high-resolution structural MRI	Estrogen receptor pathways. Interaction effects between HT and BMI on brain volume. Possible involvement of genetic variations in estrogen receptor expression.	Estrogen (specifically, the effects of HT involving CEE and other forms of exogenous estrogen).	Hormonal and hormonal replacement treatments	[53]
Ovx LDLR^−/−^ mice SH-SY5Y cells treated with PA (in vivo and in vitro)	Mouse hippocampal tissue. SH-SY5Y cells	ERα, ERβ, and GPER signaling pathways. Lipid metabolism pathways involving dyslipidemia and its effects on cognitive functions	β-Estradiol simvastatin AB23A	Hormonal and hormonal replacement treatments	[54]
Network pharmacology molecular docking (bioinformatic modeling)	Acori Graminei Rhizoma	Alzheimer’s disease pathway Serotonin synapses Estrogen signaling pathway Dopaminergic synapses PI3K-Akt signaling pathway	Acori Graminei Rhizoma	Phytocompounds	[55]
Integrated microarray analysis using gene expression datasets from the GEO database (bioinformatic modeling)	Brain tissue samples from AD patients and normal controls	Neuroactive ligand–receptor interaction Regulation of the actin cytoskeleton Estrogen signaling pathway Notch signaling pathway	XNJ	Phytocompounds	[56]
KM mice and SD rats (in vivo and in vitro)	Brain and hippocampal tissue	The estrogen signaling pathway, synaptic signaling pathway, ERα-BDNF pathway, actin cytoskeleton regulation, and MAPK signaling pathway	SCPE	Phytocompounds	[57]
Scopolamine-induced memory impairment model in mice (in vivo and in vitro)	brain tissue of mice.	The antioxidant pathway, anti-inflammatory pathway, immunomodulatory pathway, and apoptotic pathway	Silybum marianum (milk thistle)	Phytocompounds	[58]
Primary cortical neurons from rat embryos (in vivo and in vitro)	Primary cortical neurons exposed to Aβ1–42	Estrogen receptor (ER) pathway	Puerarin	Phytocompounds	[59]
Transgenic mouse model (APP/PS1 transgenic mice) (in vivo and in vitro)	Male APP/PS1 transgenic mice aged 7–8 months old. Male C57BL/6 mice of the same age as controls.	Protein kinase C pathway	Calycosin	Phytocompounds	[60]
Primary cultured hippocampal neurons from rats. (in vivo and in vitro)	Primary hippocampal neurons extracted from Sprague Dawley rats.	The non-classical estrogen signaling pathway, PI3K pathway, And MAPK pathway	Geniposide	Phytocompounds	[61]
Adult male Wistar rats ORX and treated with various doses of YCJ or EB (in vivo and in vitro)	Brains of adult male Wistar rats	NF200 PV ERα and ERβ) Aβ accumulation	YCJ, rich in β-sitosterol with estrogenic properties	Phytocompounds	[62]
Masking of endogenous estrogens and the ERβ-selective agonist DPN as boronic acid pinacol esters (chemical synthesis experiments)	Derivatives of estrone and estradiol. Non-steroidal ERβ-selective agonist DPN. Cellular environment for ERβ transcriptional activity assays.	Activation of ERα and ERβ Modulation of ERβ transcriptional activity in response to pathological H_2_O_2_ levels.	DPN, an ERβ-selective agonist.	Synthetic and non-hormonal estrogenic modulators	[63]
Middle-aged Long–Evans rats (in vivo and in vitro)	Rat hippocampus	NF-kB activation NLRP3 Inflammasome pathway Esr1 Scn1a	AbO MCC950	Synthetic and Non-Hormonal Estrogenic Modulators	[64]
miPSC-NSCs (in vitro)	Oligodendrocytes, neurons, and astrocytes	ER signaling pathway	Donepezil ICI 182780, MPP and PHTPP	Synthetic and non-hormonal estrogenic modulators	[65]
Hippocampal neurons (in vitro)	Rat hippocampal neurons	Estrogen receptor pathway	ICI 182780 (Faslodex)	Synthetic and non-hormonal estrogenic nodulators	[66]
OVX female C57BL/6J mice and nERα KO mice (in vivo and in vitro)	Brain tissue, specifically BFC neurons, NBM, and somatosensory cortex	MAPK/CREB Signaling Pathway; Components: ERK1/2, CREB; Activation: Phosphorylation of ERK1/2 and CREB in BFC neurons	4-estren-3α, 17β-diol (estren)	Synthetic and non-hormonal estrogenic modulators	[67]
Primary neocortical cultures from E15 embryos of CD-1^®^ IGS Swiss mice (in vivo and in vitro)	Mouse neocortical neurons	Apoptotic pathways (mitochondrial and external); oxidative stress pathway; MAPK and mTOR signaling pathways	PaPE-1 ((S)-5-(4-hydroxy-3,5-dimethyl-phenyl)-indan-1-ol)	Synthetic and non-hormonal estrogenic modulators	[68]
MC65 and SH-SY5Y neuroblastoma cell lines, as well as primary hippocampal neurons from WT and Tg2576 mice (in vivo and in vitro)	MC65 and SH-SY5Y and primary hippocampal neurons isolated from embryonic WT and Tg2576 mice	The protective effects of STX are mediated via GqMER, involving pathways such as ERK/MAPK, PKCδ/PKA, and PI3K signaling	STX	Synthetic and non-hormonal estrogenic modulators	[69]
Adult and pregnant Wistar rats (in vivo and in vitro)	Adult rat brains (hippocampus), fetal brains, and primary hippocampal neurons	Aβ42 accumulation, caspase-3 activity, presenilin-1 protein expression, and androgen- and estrogen receptor-mediated pathways	17β-trenbolone	Synthetic and non-hormonal estrogenic modulators	[70]
C57BL/6 (in vivo and in vitro)	The SN56 cells used are derived from murine C57BL/6 neurons, representing a model for cholinergic neuronal studies	ER signaling, Tau phosphorylation, and JNK activation	A1254 and E2	Synthetic and non-hormonal estrogenic modulators	[71]
SH-SY5Y (in vitro)	Aβo and SH-SY5Y cells	GPER, ER, oxidative stress pathways, NMDA receptor, L-type voltage-gated calcium channels, and phospholipid peroxidation pathways	Raloxifene, estradiol, fulvestrant, and G-15	Synthetic and non-hormonal estrogenic modulators	[72]

OVX: ovariectomized; DG: dentate gyrus; E2: estradiol; ESG: estrogen signaling pathway; NBM: nucleus basalis of Meynert; NGF: nerve growth factor; ERα: estrogen receptor alpha; BFC: basal forebrain cholinergic neurons; CREB: cAMP response element-binding protein; ERK: extracellular signal-regulated kinase; PaPE-1: ((S)-5-(4-hydroxy-3,5-dimethyl-phenyl)-indan-1-ol); WT: wild type; Tg2576: transgenic mouse model of Alzheimer’s disease; GqMER: G protein-coupled estrogen receptor; miPSC-NSCs: mouse induced pluripotent stem cell-derived neural stem cells; ER: estrogen receptor; SCPE: Sagacious Confucius’ Pillow Elixir; Aβo: amyloid-β oligomers; GPER: G protein-coupled estrogen receptor; NMDA: N-Methyl-D-Aspartate; APP: amyloid precursor protein; PKC: protein kinase C; NF200: neurofilament 200; PV: parvalbumin; Aβ: beta-amyloid; N27: neuronal cell line derived from rat dopaminergic neurons; PC12: cell line derived from rat adrenal pheochromocytoma; CEE: conjugated equine estrogen; XNJ: Xingnaojing; ERβ: estrogen receptor beta; PI3K: Phosphatidylinositol 3-Kinase; mER: membrane estrogen receptor; SN56: cholinergic cell line; JNK: c-Jun N-terminal kinase; A1254: Aroclor1254; CD-1^®^ IGS: Swiss mouse line used for toxicology and pharmacology studies; SH-SY5Y: human neuroblastoma cell line; MAPK: mitogen-activated protein kinase; mTOR: mechanistic target of rapamycin; Aβ: beta-amyloid; NF-κB: nuclear factor kappa B; NLRP3: NOD-like receptor family pyrin domain containing 3; Esr1: estrogen receptor 1; Scn1a: sodium channel, voltage-gated, type I, alpha Subunit; VDAC1: voltage-dependent anion channel 1; IGF-1Rb: insulin-like growth factor 1 receptor beta; APP/PS1: transgenic mouse model of Alzheimer’s disease; N27: dopaminergic neuronal cell line; AR45: androgen receptor 45; AB23A: Alisol B 23-acetate; H_2_O_2_: hydrogen peroxide.

## Data Availability

Not applicable.

## References

[1] Andrade-Moraes C.H., Oliveira-Pinto A.V., Castro-Fonseca E., da Silva C.G., Guimarães D.M., Szczupak D., Parente-Bruno D.R., Carvalho L.R., Polichiso L., Gomes B.V. (2013). Cell number changes in Alzheimer’s disease relate to dementia, not to plaques and tangles. Brain.

[2] Rahman M.M., Lendel C. (2021). Extracellular protein components of amyloid plaques and their roles in Alzheimer’s disease pathology. Mol. Neurodegener..

[3] Murray C.E., Gami-Patel P., Gkanatsiou E., Brinkmalm G., Portelius E., Wirths O., Heywood W., Blennow K., Ghiso J., Holton J.L. (2018). The presubiculum is preserved from neurodegenerative changes in Alzheimer’s disease. Acta Neuropathol. Commun..

[4] Long J.M., Holtzman D.M. (2019). Alzheimer Disease: An Update on Pathobiology and Treatment Strategies. Cell.

[5] Trejo-Lopez J.A., Yachnis A.T., Prokop S. (2022). Neuropathology of Alzheimer’s Disease. Neurotherapeutics.

[6] Montero-Crespo M., Domínguez-Álvaro M., Alonso-Nanclares L., DeFelipe J., Blazquez-Llorca L. (2021). Three-dimensional analysis of synaptic organization in the hippocampal CA1 field in Alzheimer’s disease. Brain.

[7] Uddin M.S., Rahman M.M., Jakaria M., Rahman M.S., Hossain M.S., Islam A., Ahmed M., Mathew B., Omar U.M., Barreto G.E. (2020). Estrogen Signaling in Alzheimer’s Disease: Molecular Insights and Therapeutic Targets for Alzheimer’s Dementia. Mol. Neurobiol..

[8] Tahami Monfared A.A., Byrnes M.J., White L.A., Zhang Q. (2022). Alzheimer’s Disease: Epidemiology and Clinical Progression. Neurol. Ther..

[9] Liss J.L., Seleri Assunção S., Cummings J., Atri A., Geldmacher D.S., Candela S.F., Devanand D.P., Fillit H.M., Susman J., Mintzer J. (2021). Practical recommendations for timely, accurate diagnosis of symptomatic Alzheimer’s disease (MCI and dementia) in primary care: A review and synthesis. J. Internal Med..

[10] Profenno L.A., Porsteinsson A.P., Faraone S.V. (2010). Meta-analysis of Alzheimer’s disease risk with obesity, diabetes, and related disorders. Biol. Psychiatry.

[11] Norton S., Matthews F.E., Barnes D.E., Yaffe K., Brayne C. (2014). Potential for primary prevention of Alzheimer’s disease: An analysis of population-based data. Lancet Neurol..

[12] Rusanen M., Rovio S., Ngandu T., Nissinen A., Tuomilehto J., Soininen H., Kivipelto M. (2010). Midlife smoking, apolipoprotein E and risk of dementia and Alzheimer’s disease: A population-based cardiovascular risk factors, aging and dementia study. Dement. Geriatr. Cognit. Disord..

[13] Nianogo R.A., Rosenwohl-Mack A., Yaffe K., Carrasco A., Hoffmann C.M., Barnes D.E. (2022). Risk Factors Associated With Alzheimer Disease and Related Dementias by Sex and Race and Ethnicity in the US. JAMA Neurol..

[14] Sando S.B., Melquist S., Cannon A., Hutton M.L., Sletvold O., Saltvedt I., White L.R., Lydersen S., Aasly J.O. (2008). APOE epsilon 4 lowers age at onset and is a high risk factor for Alzheimer’s disease; a case control study from central Norway. BMC Neurol..

[15] Ngandu T., Lehtisalo J., Solomon A., Levälahti E., Ahtiluoto S., Antikainen R., Bäckman L., Hänninen T., Jula A., Laatikainen T. (2015). A 2 year multidomain intervention of diet, exercise, cognitive training, and vascular risk monitoring versus control to prevent cognitive decline in at-risk elderly people (FINGER): A randomised controlled trial. Lancet.

[16] Barnes D.E., Yaffe K. (2011). The projected effect of risk factor reduction on Alzheimer’s disease prevalence. Lancet Neurol..

[17] Iso-Markku P., Kujala U.M., Knittle K., Polet J., Vuoksimaa E., Waller K. (2022). Physical activity as a protective factor for dementia and Alzheimer’s disease: Systematic review, meta-analysis and quality assessment of cohort and case–control studies. Br. J. Sports Med..

[18] Beydoun M.A., Kivimaki M. (2020). Midlife obesity, related behavioral factors, and the risk of dementia in later life. Neurology.

[19] Simpkins J.W., Perez E., Wang X., Yang S., Wen Y., Singh M. (2009). The potential for estrogens in preventing Alzheimer’s disease and vascular dementia. Therap. Adv. Neurol. Disord..

[20] Scudiero R., Verderame M. (2017). Gene expression profile of estrogen receptors alpha and beta in rat brain during aging and following high fat diet. C. R. Biol..

[21] Sharma P.K., Thakur M.K. (2006). Expression of estrogen receptor (ER) alpha and beta in mouse cerebral cortex: Effect of age, sex and gonadal steroids. Neurobiol. Aging.

[22] Maioli S., Leander K., Nilsson P., Nalvarte I. (2021). Estrogen receptors and the aging brain. Essays Biochem..

[23] Waters E.M., Yildirim M., Janssen W.G., Lou W.Y., McEwen B.S., Morrison J.H., Milner T.A. (2011). Estrogen and aging affect the synaptic distribution of estrogen receptor β-immunoreactivity in the CA1 region of female rat hippocampus. Brain Res..

[24] Mehra R.D., Sharma K., Nyakas C., Vij U. (2005). Estrogen receptor alpha and beta immunoreactive neurons in normal adult and aged female rat hippocampus: A qualitative and quantitative study. Brain Res..

[25] Lan Y.L., Zhao J., Li S. (2015). Update on the neuroprotective effect of estrogen receptor alpha against Alzheimer’s disease. J. Alzheimer’s Dis. JAD.

[26] Oveisgharan S., Yang J., Yu L., Burba D., Bang W., Tasaki S., Grodstein F., Wang Y., Zhao J., De Jager P.L. (2023). Estrogen Receptor Genes, Cognitive Decline, and Alzheimer Disease. Neurology.

[27] Ostlund H., Keller E., Hurd Y.L. (2003). Estrogen receptor gene expression in relation to neuropsychiatric disorders. Ann. N. Y. Acad. Sci..

[28] Osterlund M.K., Gustafsson J.A., Keller E., Hurd Y.L. (2000). Estrogen receptor beta (ERbeta) messenger ribonucleic acid (mRNA) expression within the human forebrain: Distinct distribution pattern to ERalpha mRNA. J. Clin. Endocrinol. Metabol..

[29] Urdinguio R.G., Sanchez-Mut J.V., Esteller M. (2009). Epigenetic mechanisms in neurological diseases: Genes, syndromes, and therapies. Lancet Neurol..

[30] Feng J., Fouse S., Fan G. (2007). Epigenetic regulation of neural gene expression and neuronal function. Pediatr. Res..

[31] Westberry J.M., Trout A.L., Wilson M.E. (2010). Epigenetic Regulation of Estrogen Receptor α Gene Expression in the Mouse Cortex during Early Postnatal Development. Endocrinology.

[32] Gong Z., Yang S., Wei M., Vlantis A.C., Chan J.Y.K., van Hasselt C.A., Li D., Zeng X., Xue L., Tong M.C.F. (2022). The Isoforms of Estrogen Receptor Alpha and Beta in Thyroid Cancer. Front. Oncol..

[33] Nagler J.J., Cavileer T., Sullivan J., Cyr D.G., Rexroad C. (2007). The complete nuclear estrogen receptor family in the rainbow trout: Discovery of the novel ERalpha2 and both ERbeta isoforms. Gene.

[34] Pace P., Taylor J., Suntharalingam S., Coombes R.C., Ali S. (1997). Human estrogen receptor beta binds DNA in a manner similar to and dimerizes with estrogen receptor alpha. J. Biol. Chem..

[35] Paterni I., Granchi C., Katzenellenbogen J.A., Minutolo F. (2014). Estrogen receptors alpha (ERα) and beta (ERβ): Subtype-selective ligands and clinical potential. Steroids.

[36] Lo R., Matthews J. (2010). A new class of estrogen receptor beta-selective activators. Mol. Interv..

[37] Nilsson S., Gustafsson J.A. (2002). Biological role of estrogen and estrogen receptors. Crit. Rev. Biochem. Mol. Biol..

[38] Dama A., Baggio C., Boscaro C., Albiero M., Cignarella A. (2021). Estrogen Receptor Functions and Pathways at the Vascular Immune Interface. Int. J. Mol. Sci..

[39] Younesi E., Hofmann-Apitius M. (2013). A network model of genomic hormone interactions underlying dementia and its translational validation through serendipitous off-target effect. J. Transl. Med..

[40] Xing F.Z., Zhao Y.G., Zhang Y.Y., He L., Zhao J.K., Liu M.Y., Liu Y., Zhang J.Q. (2018). Nuclear and membrane estrogen receptor antagonists induce similar mTORC2 activation-reversible changes in synaptic protein expression and actin polymerization in the mouse hippocampus. CNS Neurosci. Ther..

[41] Xiong Y.S., Liu F.F., Liu D., Huang H.Z., Wei N., Tan L., Chen J.G., Man H.Y., Gong C.X., Lu Y. (2015). Opposite effects of two estrogen receptors on tau phosphorylation through disparate effects on the miR-218/PTPA pathway. Aging Cell.

[42] Wang C., Zhang F., Jiang S., Siedlak S.L., Shen L., Perry G., Wang X., Tang B., Zhu X. (2016). Estrogen receptor-α is localized to neurofibrillary tangles in Alzheimer’s disease. Sci. Rep..

[43] Mesa-Herrera F., Marín R., Torrealba E., Santos G., Díaz M. (2022). Neuronal ER-Signalosome Proteins as Early Biomarkers in Prodromal Alzheimer’s Disease Independent of Amyloid-β Production and Tau Phosphorylation. Front. Mol. Neurosci..

[44] Buchhave P., Minthon L., Zetterberg H., Wallin A.K., Blennow K., Hansson O. (2012). Cerebrospinal fluid levels of β-amyloid 1-42, but not of tau, are fully changed already 5 to 10 years before the onset of Alzheimer dementia. Arch. Gen. Psychiatry.

[45] Chiu M.J., Chen T.F., Hu C.J., Yan S.H., Sun Y., Liu B.H., Chang Y.T., Yang C.C., Yang S.Y. (2020). Nanoparticle-based immunomagnetic assay of plasma biomarkers for differentiating dementia and prodromal states of Alzheimer’s disease—A cross-validation study. Nanomed. Nanotechnol. Biol. Med.

[46] Wang X.L., Li L. (2021). Cell type-specific potential pathogenic genes and functional pathways in Alzheimer’s Disease. BMC Neurol..

[47] Ishunina T.A., Swaab D.F. (2021). Estrogen receptor α splice variant TADDI in the human supraoptic nucleus: An effect on neuronal size and changes in pneumonia. Neuro Endocrinol. Lett..

[48] Iacobas D.A., Iacobas S., Nebieridze N., Velíšek L., Velíšková J. (2018). Estrogen Protects Neurotransmission Transcriptome During Status Epilepticus. Front. Neurosci..

[49] Morelli A., Sarchielli E., Guarnieri G., Coppi E., Pantano D., Comeglio P., Nardiello P., Pugliese A.M., Ballerini L., Matucci R. (2017). Young Human Cholinergic Neurons Respond to Physiological Regulators and Improve Cognitive Symptoms in an Animal Model of Alzheimer’s Disease. Front. Cell. Neurosci..

[50] Pooley A.E., Luong M., Hussain A., Nathan B.P. (2015). Neurite outgrowth promoting effect of 17-β estradiol is mediated through estrogen receptor alpha in an olfactory epithelium culture. Brain Res..

[51] Duong P., Tenkorang M.A.A., Trieu J., McCuiston C., Rybalchenko N., Cunningham R.L. (2020). Neuroprotective and neurotoxic outcomes of androgens and estrogens in an oxidative stress environment. Biol. Sex Differ..

[52] Canerina-Amaro A., Hernandez-Abad L.G., Ferrer I., Quinto-Alemany D., Mesa-Herrera F., Ferri C., Puertas-Avendano R.A., Diaz M., Marin R. (2017). Lipid raft ER signalosome malfunctions in menopause and Alzheimer’s disease. Front. Biosci..

[53] Boyle C.P., Raji C.A., Erickson K.I., Lopez O.L., Becker J.T., Gach H.M., Kuller L.H., Longstreth W., Carmichael O.T., Riedel B.C. (2021). Estrogen, brain structure, and cognition in postmenopausal women. Hum. Brain Mapp..

[54] Meng Q., Chao Y., Zhang S., Ding X., Feng H., Zhang C., Liu B., Zhu W., Li Y., Zhang Q. (2023). Attenuation of estrogen and its receptors in the post-menopausal stage exacerbates dyslipidemia and leads to cognitive impairment. Mol. Brain.

[55] Du Y.K., Xiao Y., Zhong S.M., Huang Y.X., Chen Q.W., Zhou Y.Q., Guo J.Y., Yang C. (2021). Study on the Mechanism of Acori Graminei Rhizoma in the Treatment of Alzheimer’s Disease Based on Network Pharmacology and Molecular Docking. BioMed Res. Int..

[56] Wang M., Wang S., Li Y., Cai G., Cao M., Li L. (2020). Integrated analysis and network pharmacology approaches to explore key genes of Xingnaojing for treatment of Alzheimer’s disease. Brain Behav..

[57] Zhao D.P., Lei X., Wang Y.Y., Xue A., Zhao C.Y., Xu Y.M., Zhang Y., Liu G.L., Geng F., Xu H.D. (2022). Sagacious confucius’ pillow elixir ameliorates Dgalactose induced cognitive injury in mice via estrogenic effects and synaptic plasticity. Front. Pharmacol..

[58] Song X., Liu B., Cui L., Zhou B., Liu L., Liu W., Yao G., Xia M., Hayashi T., Hattori S. (2018). Estrogen Receptors Are Involved in the Neuroprotective Effect of Silibinin in Aβ(1-42)-Treated Rats. Neurochem. Res..

[59] Li L., Xue Z., Chen L., Chen X., Wang H., Wang X. (2017). Puerarin suppression of Aβ(1-42)-induced primary cortical neuron death is largely dependent on ERβ. Brain Res..

[60] Song L., Li X., Bai X.X., Gao J., Wang C.Y. (2017). Calycosin improves cognitive function in a transgenic mouse model of Alzheimer’s disease by activating the protein kinase C pathway. Neural Regener. Res..

[61] Li J., Wang F., Ding H., Jin C., Chen J., Zhao Y., Li X., Chen W., Sun P., Tan Y. (2014). Geniposide, the component of the Chinese herbal formula Tongluojiunao, protects amyloid-β peptide (1-42-mediated death of hippocampal neurons via the non-classical estrogen signaling pathway. Neural Regener. Res..

[62] Balit T., Abdel-Wahhab M.A., Radenahmad N. (2019). Young Coconut Juice Reduces Some Histopathological Changes Associated with Alzheimer’s Disease through the Modulation of Estrogen Receptors in Orchidectomized Rat Brains. J. Aging Res..

[63] Park H., McEachon J.D., Pollock J.A. (2019). Synthesis and characterization of hydrogen peroxide activated estrogen receptor beta ligands. Bioorg. Med. Chem..

[64] Fekete C., Vastagh C., Dénes Á., Hrabovszky E., Nyiri G., Kalló I., Liposits Z., Sárvári M. (2019). Chronic Amyloid β Oligomer Infusion Evokes Sustained Inflammation and Microglial Changes in the Rat Hippocampus via NLRP3. Neuroscience.

[65] Imamura O., Arai M., Dateki M., Oishi K., Takishima K. (2020). Donepezil-induced oligodendrocyte differentiation is mediated through estrogen receptors. J. Neurochem..

[66] Zhao L., O’Neill K., Brinton R.D. (2006). Estrogenic agonist activity of ICI 182780 (Faslodex) in hippocampal neurons: Implications for basic science understanding of estrogen signaling and development of estrogen modulators with a dual therapeutic profile. J. Pharmacol. Exp. Therap..

[67] Kwakowsky A., Potapov K., Kim S., Peppercorn K., Tate W.P., Ábrahám I.M. (2016). Treatment of beta amyloid 1-42 (Aβ(1-42))-induced basal forebrain cholinergic damage by a non-classical estrogen signaling activator in vivo. Sci. Rep..

[68] Wnuk A., Przepiórska K., Rzemieniec J., Pietrzak B., Kajta M. (2020). Selective Targeting of Non-nuclear Estrogen Receptors with PaPE-1 as a New Treatment Strategy for Alzheimer’s Disease. Neurotox. Res..

[69] Gray N.E., Zweig J.A., Kawamoto C., Quinn J.F., Copenhaver P.F. (2016). STX, a Novel Membrane Estrogen Receptor Ligand, Protects Against Amyloid-β Toxicity. J. Alzheimer’s Dis. JAD.

[70] Ma F., Liu D. (2015). 17β-trenbolone, an anabolic-androgenic steroid as well as an environmental hormone, contributes to neurodegeneration. Toxicol. Appl. Pharmacol..

[71] Bang Y., Lim J., Kim S.S., Jeong H.M., Jung K.K., Kang I.H., Lee K.Y., Choi H.J. (2011). Aroclor1254 interferes with estrogen receptor-mediated neuroprotection against beta-amyloid toxicity in cholinergic SN56 cells. Neurochem. Int..

[72] Nohara T., Tsuji M., Oguchi T., Momma Y., Ohashi H., Nagata M., Ito N., Yamamoto K., Murakami H., Kiuchi Y. (2023). Neuroprotective Potential of Raloxifene via G-Protein-Coupled Estrogen Receptors in Aβ-Oligomer-Induced Neuronal Injury. Biomedicines.

